# Composition of the Survival Motor Neuron (SMN) Complex in *Drosophila melanogaster*

**DOI:** 10.1534/g3.118.200874

**Published:** 2018-12-21

**Authors:** A. Gregory Matera, Amanda C. Raimer, Casey A. Schmidt, Jo A. Kelly, Gaith N. Droby, David Baillat, Sara ten Have, Angus I. Lamond, Eric J. Wagner, Kelsey M. Gray

**Affiliations:** * Integrative Program for Biological and Genome Sciences; † Curriculum in Genetics and Molecular Biology; ‡ Lineberger Comprehensive Cancer Center; § Department of Biology; ** Department of Genetics, The University of North Carolina, Chapel Hill, NC 27599; †† Department of Biochemistry and Molecular Biology, University of Texas Medical Branch, Galveston, TX 77550; ‡‡ Centre for Gene Regulation and Expression, School of Life Sciences, University of Dundee, Dundee, DD15EH, UK

**Keywords:** locomotor function, ncRNA, proteomics, RNP assembly, SMN, survival motor neuron, snRNA, snRNP, Spinal Muscular Atrophy, SMA

## Abstract

Spinal Muscular Atrophy (SMA) is caused by homozygous mutations in the human *survival motor neuron 1* (*SMN1*) gene. SMN protein has a well-characterized role in the biogenesis of small nuclear ribonucleoproteins (snRNPs), core components of the spliceosome. SMN is part of an oligomeric complex with core binding partners, collectively called Gemins. Biochemical and cell biological studies demonstrate that certain Gemins are required for proper snRNP assembly and transport. However, the precise functions of most Gemins are unknown. To gain a deeper understanding of the SMN complex in the context of metazoan evolution, we investigated its composition in *Drosophila melanogaster*. Using transgenic flies that exclusively express Flag-tagged SMN from its native promoter, we previously found that Gemin2, Gemin3, Gemin5, and all nine classical Sm proteins, including Lsm10 and Lsm11, co-purify with SMN. Here, we show that CG2941 is also highly enriched in the pulldown. Reciprocal co-immunoprecipitation reveals that epitope-tagged CG2941 interacts with endogenous SMN in Schneider2 cells. Bioinformatic comparisons show that CG2941 shares sequence and structural similarity with metazoan Gemin4. Additional analysis shows that three other genes (*CG14164*, *CG31950* and *CG2371*) are not orthologous to Gemins 6-7-8, respectively, as previously suggested. In *D.melanogaster*, *CG2941* is located within an evolutionarily recent genomic triplication with two other nearly identical paralogous genes (*CG32783* and *CG32786*). RNAi-mediated knockdown of *CG2941* and its two close paralogs reveals that *Gemin4* is essential for organismal viability.

Spinal muscular atrophy (SMA) is a pediatric neuromuscular disorder caused by mutation or loss of the human *survival motor neuron 1* (*SMN1*) gene (Lefebvre *et al.* 1995). Approximately 95% of SMA patients have homozygous deletions in *SMN1*, and the remaining ∼5% are hemizygous for the deletion over a missense mutation in *SMN1* (Burghes and Beattie 2009). Despite the great progress that has been made therapeutically, the etiology of SMA remains poorly understood. SMN’s best understood function is in the biogenesis of spliceosomal uridine-rich small nuclear ribonucleoproteins (UsnRNPs), a fundamental process important for all eukaryotic cells (Battle *et al.* 2006a; Matera *et al.* 2007; Coady and Lorson 2011; Fischer *et al.* 2011; Matera and Wang 2014). Additional tissue-specific functions for SMN have also been reported (for reviews, see Fallini *et al.* 2012; Hamilton and Gillingwater 2013; Shababi *et al.* 2014; Nash *et al.* 2016; Chaytow *et al.* 2018).

To date, no definitive link has been established between a specific function of SMN and SMA pathogenesis. Moving forward, the key question in the field is to identify which of the many potential activities of SMN lie at the root of the neuromuscular dysfunction. Given that SMA is a spectrum disorder with a broad range of phenotypes (Tizzano and Finkel 2017; Groen *et al.* 2018), it seems likely that the most severe forms of the disease would involve loss of more than one SMN-dependent pathway. Thus, understanding the molecular etiology of the disease is not only important for the basic biology, but also for targeting and refining therapeutic strategies (Groen *et al.* 2018; Sumner and Crawford 2018).

As outlined in Figure 1, SMN works in close partnership with a number of proteins, collectively called Gemins (Paushkin *et al.* 2002; Shpargel and Matera 2005; Otter *et al.* 2007; Borg and Cauchi 2013). The N-terminal domain of SMN interacts with a protein called Gemin2 (Liu *et al.* 1997; Wang and Dreyfuss 2001), whereas the C-terminal region contains a YG-zipper motif (Martin *et al.* 2012) that drives SMN self-oligomerization, as well as binding to Gemin3 (Lorson *et al.* 1998; Pellizzoni *et al.* 1999; Praveen *et al.* 2014; Gupta *et al.* 2015). Gemin2 heterodimerizes with SMN and is the only member of the complex that is conserved from budding yeast to humans (Fischer *et al.* 1997; Kroiss *et al.* 2008). When provided with *in vitro* transcribed Sm-class snRNAs, purified recombinant SMN and Gemin2 are sufficient for Sm core assembly activity (Kroiss *et al.* 2008). *In vivo*, Gemin5 is thought to play an important role in snRNA substrate recognition (Battle *et al.* 2006b; Bradrick and Gromeier 2009; Lau *et al.* 2009; Yong *et al.* 2010; Jin *et al.* 2016), but it has also been independently identified as a cellular signaling factor (Gates *et al.* 2004; Kim *et al.* 2007) and a translation factor (reviewed in Piñeiro *et al.* 2015).

**Figure 1:**
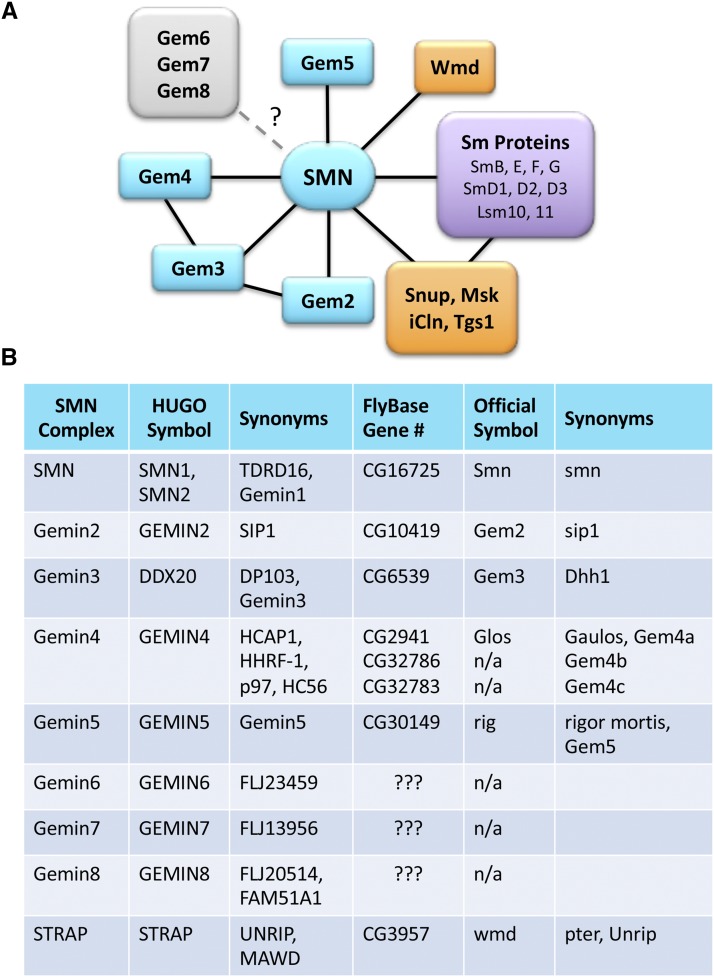
Protein interaction network of the metazoan SMN complex. (A) In addition to the core Gemin family members (shown in teal), SMN is known to form complexes with various Sm protein substrates (purple) and RNP biogenesis factors (orange). Snup, Msk, iCln, Tgs1 and Wmd, correspond to SPN1/Snurportin1, Moleskin/Importin7, CLNS1A/Chloride channel nucleotide-sensitive 1A, TGS1/Trimethylguanosine synthase 1, and STRAP/Unrip, respectively. The presence of three of the Gemins (Gem6, Gem7 and Gem8) within dipteran genomes is uncertain (?). (B) Reference table comparing the names, symbols and synonyms of SMN complex members in humans and flies.

Gemin3/DDX20/Dp103 is a DEAD-box helicase (Charroux *et al.* 1999; Grundhoff *et al.* 1999) that interacts with the SMN•Gemin2 hetero-oligomer and is reported to play roles in transcriptional repression (Yan *et al.* 2003) and microRNA activity (Mourelatos *et al.* 2002) in addition to its role in Sm-core assembly (Shpargel and Matera 2005), reviewed in (Curmi and Cauchi 2018). Gemin4 is tethered to the SMN complex via direct binding to Gemin3 (Charroux *et al.* 2000; Meister *et al.* 2000). Gemin4 has been implicated in nuclear receptor binding (Di *et al.* 2003; Yang *et al.* 2015), microRNA biology (Hutvágner and Zamore 2002; Meister *et al.* 2005) and in nuclear import of the SMN complex (Narayanan *et al.* 2004; Meier *et al.* 2018). In human cell lysates, SMN and Gemins2-4 are thought to be essential for proper assembly of the Sm core (Shpargel and Matera 2005). Consistent with this notion, complete loss-of-function mutations in *Smn*, *Gemin2*, *Gemin3* and *Gemin4* in mice result in embryonic lethality (Schrank *et al.* 1997; Jablonka *et al.* 2002; Mouillet *et al.* 2008; Meier *et al.* 2018). Partial loss-of-function mutations in genes encoding the Gemins have not been reported. Functions of the other members of the SMN complex are largely unknown.

Gemins6-8 (Gem6-7-8) and STRAP (serine-threonine kinase receptor associated protein, a.k.a. UNRIP, UNR-interacting protein; or Wmd/CG3957, wing morphogenesis defect) are peripheral members of the SMN complex and may thus serve in a regulatory capacity. In support of this idea, STRAP/UNRIP is found in a separate complex with a cap-independent translation factor called UNR (Hunt *et al.* 1999)and has been shown to modulate its function (Carissimi *et al.* 2005; Grimmler *et al.* 2005). STRAP binds to the SMN complex via an interaction with Gemin7 (Otter *et al.* 2007). In *Drosophila*, mutations in the STRAP ortholog, *Wmd/CG3957*, cause defects in development of the adult wing (Khokhar *et al.* 2008), suggesting that this protein may not be essential for basal assembly of spliceosomal snRNPs. Gem6-7-8 forms a subcomplex that is tethered to SMN via interaction with Gemin8 (Carissimi *et al.* 2006; Otter *et al.* 2007). Gemin6 and Gemin7 are Sm-like proteins that heterodimerize with one another, but the roles played by these factors in snRNP biogenesis are unknown. Mutations in Gem6-7-8 have yet to be described.

In *Drosophila*, the SMN complex (as defined by proteins that stably co-purify with SMN) was originally thought to contain only *Gemin2* and *Gemin3* (Kroiss *et al.* 2008). Bioinformatic analysis suggested that the gene *rigor mortis (Rig)* encodes a potential metazoan *Gemin5* ortholog, but Gemin5/Rig protein failed to co-purify with SMN in Schneider2 (S2) cells (Kroiss *et al.* 2008). Notably, transgenic expression of tagged *Gemin2* and *Gemin5/Rig* constructs showed that these two proteins colocalize with endogenous SMN in cytoplasmic structures called U bodies (Cauchi *et al.* 2010). Consistent with the cytological studies, we found that *Gemin5/Rig* co-purified with SMN in *Drosophila* embryos that were engineered to exclusively express Flag-tagged SMN (Gray *et al.* 2018). However, *Drosophila* Gem6-7-8 proteins were neither identified bioinformatically nor were they shown to biochemically coprecipitate with SMN in S2 cells (Kroiss *et al.* 2008).

A recent study presented evidence suggesting the “full conservation” of the SMN complex in fruit flies (Lanfranco *et al.* 2017), and arguing that Gemin4 and Gemins6-7-8 are present in Diptera. In this report, we re-investigate this issue, showing that among the four novel factors identified by Lanfranco *et al.* (2017), only *Gaulos (CG2941)* is orthologous to a metazoan SMN complex protein (Gemin4). Using comparative genomic analysis, we conclusively demonstrate that *Hezron* *(CG14164), Sabbat (CG31950)* and *Valette (CG2371)* are not orthologous to metazoan *Gemin6*, *Gemin7* and *Gemin8*, respectively. The genes encoding these three *Drosophila* proteins are actually orthologous to three distinct and highly conserved metazoan genes. The implications of these findings are discussed. Furthermore, we purified SMN complexes from *Drosophila* embryos and found that endogenous Gaulos co-precipitates with SMN but Valette, Sabbat and Hezron do not. Phylogenetic analysis demonstrates that *Gaulos/CG2941* is actually part of a genomic triplication involving two other nearly identical gene copies, *CG32783* and *CG32786*. We interrogate the function of these three factors *in vivo* using RNA interference analysis, revealing that the function of these redundant genes is essential in flies.

## Materials and Methods

### Fly stocks

RNAi lines were obtained from the Bloomington TRIP collection. The identifying numbers listed on the chart are stock numbers. Each of the RNAi constructs is expressed from one of five VALIUM vectors and requires Gal4 for expression. All stocks were cultured on molasses and agar at room temperature (25°).

### Antibodies and Western blotting

Embryonic lysates were prepared by crushing the animals in lysis buffer (50mM Tris-HCl, pH 7.5, 150 mM NaCl, 1mM EDTA, 1% NP-40) with 1X protease inhibitor cocktail (Invitrogen) and clearing the lysate by centrifugation at 13,000 RPM for 10 min at 4°. S2 cell lysates were prepared by suspending cells in lysis buffer (50mM Tris-HCl, pH 7.5, 150 mM NaCl, 1mM EDTA, 1% NP-40) with 10% glycerol and 1x protease inhibitor cocktail (Invitrogen) and disrupting cell membranes by pulling the suspension through a 25 gauge needle (Becton Dickinson). The lysate was then cleared by centrifugation at 13,000 RPM for 10 min at 4°. Cell fractionation was performed using a standard protocol (West *et al.* 2008). In brief, following centrifugation, cytoplasmic extracts were taken from the top 0.2mL and the nuclear pellet was resuspended in 0.2mL RIPA buffer. Western blotting on lysates was performed using standard protocols. Rabbit anti-dSMN serum was generated by injecting rabbits with purified, full-length dSMN protein (Pacific Immunology Corp, CA), and was subsequently affinity purified. For Western blotting, dilutions of 1 in 2,500 for the affinity purified anti-dSMN, 1 in 10,000 for monoclonal anti-FLAG (Sigma) were used.

### Immunoprecipitation

Lysates were incubated with Anti-FLAG antibody crosslinked to agarose beads (EZview Red Anti-FLAG M2 affinity gel, Sigma) for 2h-ON at 4C with rotation. The beads were washed with RIPA lysis buffer or three times and boiled in SDS gel-loading buffer. Eluted proteins were run on an SDS-PAGE for western blotting.

### Drosophila embryo protein lysate and mass spectrometry

0-12h *Drosophila* embryos were collected from Oregon-R control and Flag-SMN flies, dechorionated, flash frozen, and stored at -80°. Embryos (approx. 1gr) were then homogenized on ice with a Potter tissue grinder in 5 mL of lysis buffer containing 100mM potassium acetate, 30mM HEPES-KOH at pH 7.4, 2mM magnesium acetate, 5mM dithiothreitol (DTT) and protease inhibitor cocktail. Lysates were centrifuged twice at 20,000 rpm for 20min at 4° and dialyzed for 5h at 4° in Buffer D (HEPES 20mM pH 7.9, 100mM KCl, 2.5 mM MgCl_2_, 20% glycerol, 0.5 mM DTT, PMSF 0.2 mM). Lysates were clarified again by centrifugation at 20000 rpm for 20 min at 4C. Lysates were flash frozen using liquid nitrogen and stored at -80C before use. Lysates were then thawed on ice, centrifuged at 20000 rpm for 20 min at 4C and incubated with rotation with 100 uL of EZview Red Anti-FLAG M2 affinity gel (Sigma) for 2h at 4C. Beads were washed a total of six times using buffer with KCl concentrations ranging from 100mM to 250mM with rotation for 1 min at 4° in between each wash. Finally, Flag proteins were eluted 3 consecutive times with one bed volume of elution buffer (Tris 20mM pH 8, 100 mM KCl, 10% glycerol, 0.5 mM DTT, PMSF 0.2 mM) containing 250ug/mL 3XFLAG peptide (sigma). The eluates were used for mass spectrometric analysis on an Orbitrap Velos instrument, fitted with a Thermo Easy-spray 50cm column.

### Viability and Larval Locomotion

Males containing RNAi constructs were crossed to virgin females containing one of the Gal4 constructs balanced by CAG (*Tub-Gal4*) or TM6BGFP (*Da-Gal4*). Embryos were collected on molasses agar plates and sorted into vials using lack of GFP fluorescence. A maximum of 50 larvae were sorted into each vial. Viability was assessed based on the number of pupated or eclosed individuals compared to the starting number of larvae in each vial.

To assess larval locomotion, five wandering third instar larvae were set on a large molasses agar plate and placed in a recording chamber. Their crawling movements were recorded for at least 1 min on a digital camera (smartphone) at minimum zoom. Four recording were take for each set of larvae; at least 30 larvae total were recorded for each cross. The videos were transferred to a PC and converted to AVI files using ffmpeg (https://www.ffmpeg.org/). The videos were then opened and converted to binary frames using Fiji/ImageJ. The wrMTrck plugin (http://www.phage.dk/plugins/wrmtrck.html) for ImageJ was used to assess the average speed of each larvae normalized to their body size (body lengths/second or BLPS).

### Northern blotting and RT-PCR

Early third instar larvae (73-77 hr post egg-laying) were homogenized in TRIzol reagent (Invitrogen) and RNA was isolated according to the manufacturer’s protocol with the following modifications: a second chloroform extraction was performed, and RNA was precipitated with 0.1 volumes of sodium acetate and 2.5 volumes of ethanol rather than isopropanol. For Northern blotting, 2500 ng of total RNA was separated on Novex 10% TBE-Urea gels (Invitrogen). RNA was transferred to GeneScreen Plus Hybridization Transfer Membrane (PerkinElmer). Blots were dried, UV cross-linked, and pre-hybridized with Rapid-hyb Buffer (GE Healthcare). Probes were prepared by 5′-end labeling oligonucleotides with [γ-^32^P]ATP (PerkinElmer) using T4 PNK (NEB).

The oligonucleotide probe sequences are as follows:

U1: 5′-GAATAATCGCAGAGGTCAACTCAGCCGAGGT-3′
U2: 5′-TCCGTCTGATTCCAAAAATCAGTTTAACATTTGTTGTCCTCCAAT-3′
U4: 5′-GGGGTATTGGTTAAAGTTTTCAACTAGCAATAATCGCACCTCAGTAG-3′
U5: 5′-GACTCATTAGAGTGTTCCTCTCCACGGAAATCTTTAGTAAAAGGC-3′
U6: 5′-CTTCTCTGTATCGTTCCAATTTTAGTATATGTTCTGCCGAAGCAAGA-3′
U11: 5′-TCGTGATCGGAAACGTGCCAGGACG-3′
U12: 5′-GCCTAGAAGCCAATACTGCCAAGCGATTAGCAAG-3′
U4atac: 5′-AGCAATGTCCTCACTAGACGTTCATTGAACATTTCTGCT-3′
U6atac: 5′-CCTAGCCGACCGTTTATGTGTTCCATCCTTGTCT-3′

Following the PNK reaction, probes were purified using Microspin G-50 Columns (GE Healthcare). For hybridization, the blots were probed with the labeled oligonucleotides at 65°. The blots were then washed twice each in 2X SSC and 0.33X SSC at 60°. Blots were exposed to storage phosphor screens (GE Healthcare) and analyzed with an Amersham Typhoon 5 (GE Healthcare).

To analyze knockdown efficiency, total RNA was treated with TURBO DNase (Invitrogen). Following a phenol/chloroform purification, 350 ng of RNA was converted to cDNA using the SuperScript III First-Strand Synthesis System (Invitrogen). Primers for PCR are as follows:

5S_F: 5′-GCCAACGACCATACCACGCTGAA-3′
5S_R: 5′-AACAACACGCGGTGTTCCCAAGC -3′
CG2941_F: 5′- TGTGGTATTGGCAGGACGGTCT-3′
CG2941_R: 5′- CCTTGTGCTTCAATTTGCTCACTTGGTT -3′
G4a-b-c_F: 5′- CCAGATAGCCTGCATGGAACATCG -3′
G4a-b-c_R: 5′- CTCCCGCTTTAATGGATCATTGAGGG -3′

### Data availability

All fly strains, probe sequences and plasmids are available upon request. The authors affirm that all data necessary for confirming the conclusions of the article are present within the article, figures, and tables. Supplemental material available at Figshare: https://doi.org/10.25387/g3.7473359.

## Results and Discussion

To identify *Drosophila melanogaster* proteins that might correspond to those known to be contained within the human SMN complex (Figure 1B), we first carried out *in silico* bioinformatic analyses. As mentioned above, there have been conflicting reports regarding the conservation (or lack thereof) of certain Gemin proteins in *Drosophila* (Kroiss *et al.* 2008; Lanfranco *et al.* 2017). In particular, Gemin4, Gemin6, Gemin7 and Gemin8 were originally thought to have been lost from Dipteran genomes, although clear orthologs of Gem6-7-8 can been readily identifed in Hymenoptera, and other insects (*e.g.*, search https://www.ncbi.nlm.nih.gov/protein).

### Hezron (CG14164) is orthologous to Lsm12, not Gemin6

**Figure 2:**
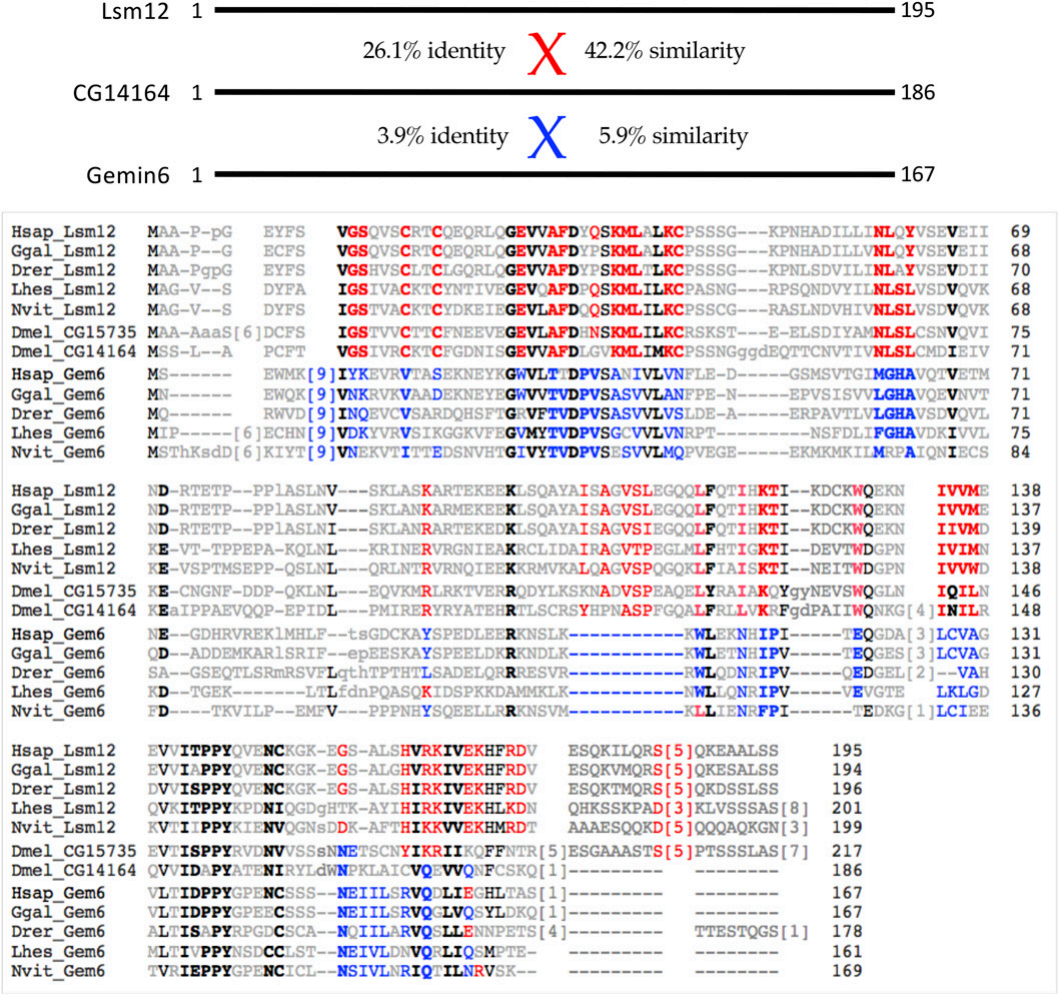
CG14164/Hezron is orthologous to Lsm12, not Gemin6. Top: Schematic of human Lsm12 and Gemin6, with pairwise alignment scores to CG14164 showing %similarity and %identity. Bottom: Amino acid alignment of Lsm12 and Gemin6 (Gem6) protein sequences from a variety of metazoan species, including: *Homo sapiens* (Hsap), *Gallus gallus* (Ggal), *Danio rerio* (Drer), *Lygus hesperus* (Lhes), and *Nasonia vitripennis* (Nvit). Two paralogous *D. melanogaster* proteins CG15735 and CG14164, are shown for comparison. Each of the fruitfly proteins shows a high degree of similarity to Lsm12, as compared to Gemin6. Diagnostic residues shaded in red are highly conserved in Lsm12 orthologs and those shaded in blue are conserved in the Gem6 orthologs. Residues bolded in black are conserved among all of the proteins.

Lanfranco *et al.* (2017) recently suggested that *Hezron/CG14164* encodes the *Drosophila* ortholog of Gemin6. Our bioinformatic analysis suggested that *CG14164* is actually more closely related to human Lsm12, an Sm-like protein that contains a C-terminal methyltransferase domain (Albrecht and Lengauer 2004). Because Gemin6 is also an Sm-like protein (Gemin6, Gemin7 and Lsm12 are members of the Sm protein superfamily), we carried out a side-by-side comparison of both Lsm12 and Gemin6 proteins from a variety of vertebrates and invertebrates (Figure 2). That is, we selected Lsm12 and Gemin6 protein pairs from each of five different species (human, chicken, fish, bug and wasp) and aligned them together to identify highly conserved diagnostic amino acid residues in each orthology cluster. Notably, there are two very closely related proteins in *D. melanogaster*, CG14164 and CG15735, both of which were included in this comparison. As shown in Figure 2, CG14164/Hezron is clearly more closely related to the Lsm12 cluster than it is to that of Gemin6 (compare diagnostic Lsm12 and Gemin6 residues shaded in red and blue, respectively). In nearly every case, CG14164 tracks with the Lsm12 sequences, including the locations of conserved insertions and deletions. Therefore, we conclude that *Hezron/CG14164* is orthologous to metazoan *Lsm12* and that the ancestral *Gemin6* gene has been lost in *Drosophila*.

*CG15735* was recently shown to function as an Ataxin-2 adaptor (Lee *et al.* 2017); a genetic analysis of *CG14164* has not been reported. We note that CG15735/Lsm12a (217aa) is slightly longer than Hezron/CG14164/Lsm12b (186aa) and that the two proteins begin to diverge only at their respective C-termini (Figure 2). There is the barest hint of similarity between CG14164 and Gemin6 in this region. It is tempting to speculate that an ancestral recombination between *Gemin6* and *Lsm12* might have created *Hezron/CG14164*. Additional experiments will be required in order to address these evolutionary relationships, as well as to determine whether or not Hezron/CG14164/Lsm12b protein might have been co-opted into the extant *Drosophila* SMN complex (see below).

### Sabbat (CG31950) is orthologous to Naa38, not Gemin7

**Figure 3:**
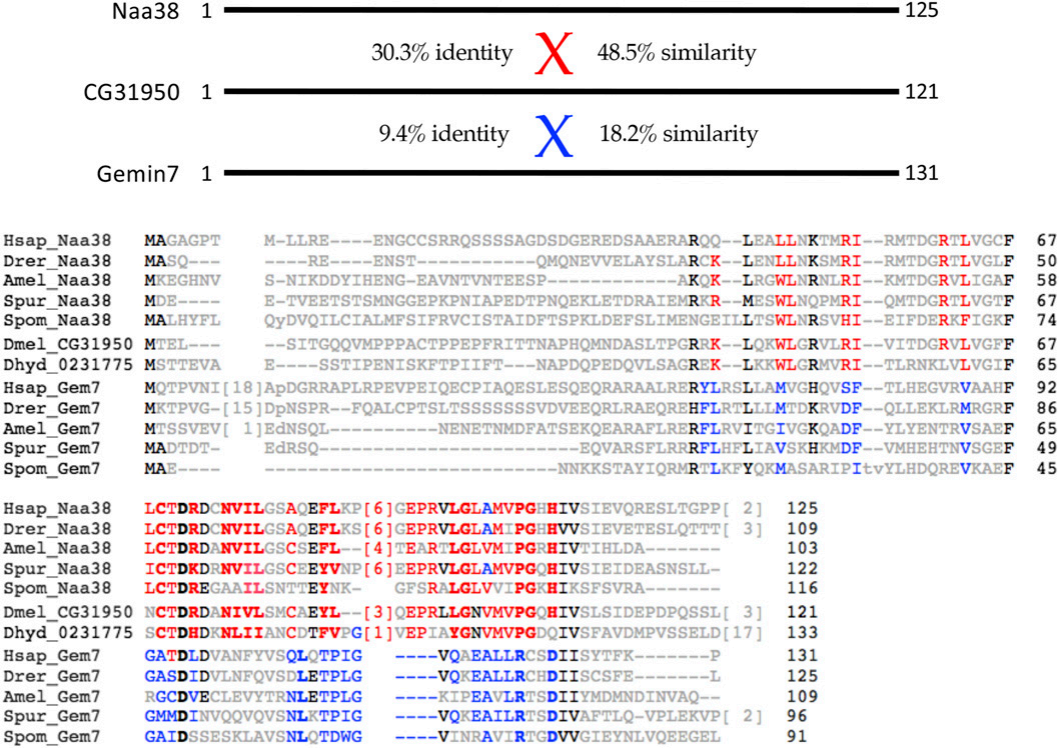
CG31950/Sabbat is orthologous to Naa38, not Gemin7. Top: Schematic of human Naa38 and Gemin7, with pairwise alignment scores to CG31950 showing %similarity and %identity. Bottom: Amino acid alignment of Gemin7 (Gem7) and Naa38 protein sequences from a variety of metazoan species, including: *Homo sapiens* (Hsap), *Danio rerio* (Drer), *Apis mellifera* (Amel), *Strongylocentrotus purpuratus* (Spur) and *Schizosaccharomyces pombe* (Spom). Two orthologous fruitfly proteins from *D. melanogaster* (Dmel_CG31950) and *D. hydei* (Dhyd_0231775) are shown for comparison. Diagnostic residues in red are those that are conserved in Naa38 sequences and those in blue are conserved in Gemin7 proteins.

*CG31950*/*Sabbat* was also identified by Lanfranco *et al.* (2017) as a potential ortholog to *Gemin7* and member of the *Drosophila* SMN complex. We found that *CG31950* was, in fact, more similar to an N-terminal acetyltransferase auxiliary subunit, *Naa38* (Varland *et al.* 2015; Aksnes *et al.* 2016). An alignment of Naa38 proteins from human, fish, honeybee, sea urchin and fission yeast, along with the Gemin7 orthologs from these same five species reveals that CG31950 is much more similar to the Naa38 orthology cluster than it is to that of Gemin7 (Figure 3). For purposes of comparison, we also include in the alignment the most closely related protein in a second fruitfly genome, *D. hydei* (XP_023177506.1), along with CG31950 from *D. melanogaster* (Figure 3). Although the two clusters of proteins share an overall sequence similarity (indeed, human Naa38 is also known to contain an Sm-like fold; see https://www.uniprot.org/uniprot/I3L310) diagnostic residues within the *Naa38* orthologs are shared by *CG31950*. In contrast, the highly conserved regions of Gemin7, including the relative positions of insertions and deletions, do not track with CG31950 (see shaded residues in Figure 3). Hence, we conclude that *Sabbat/CG31950* is orthologous to *Naa38*.

### Valette (CG2371) is orthologous to CommD10, not Gemin8

**Figure 4:**
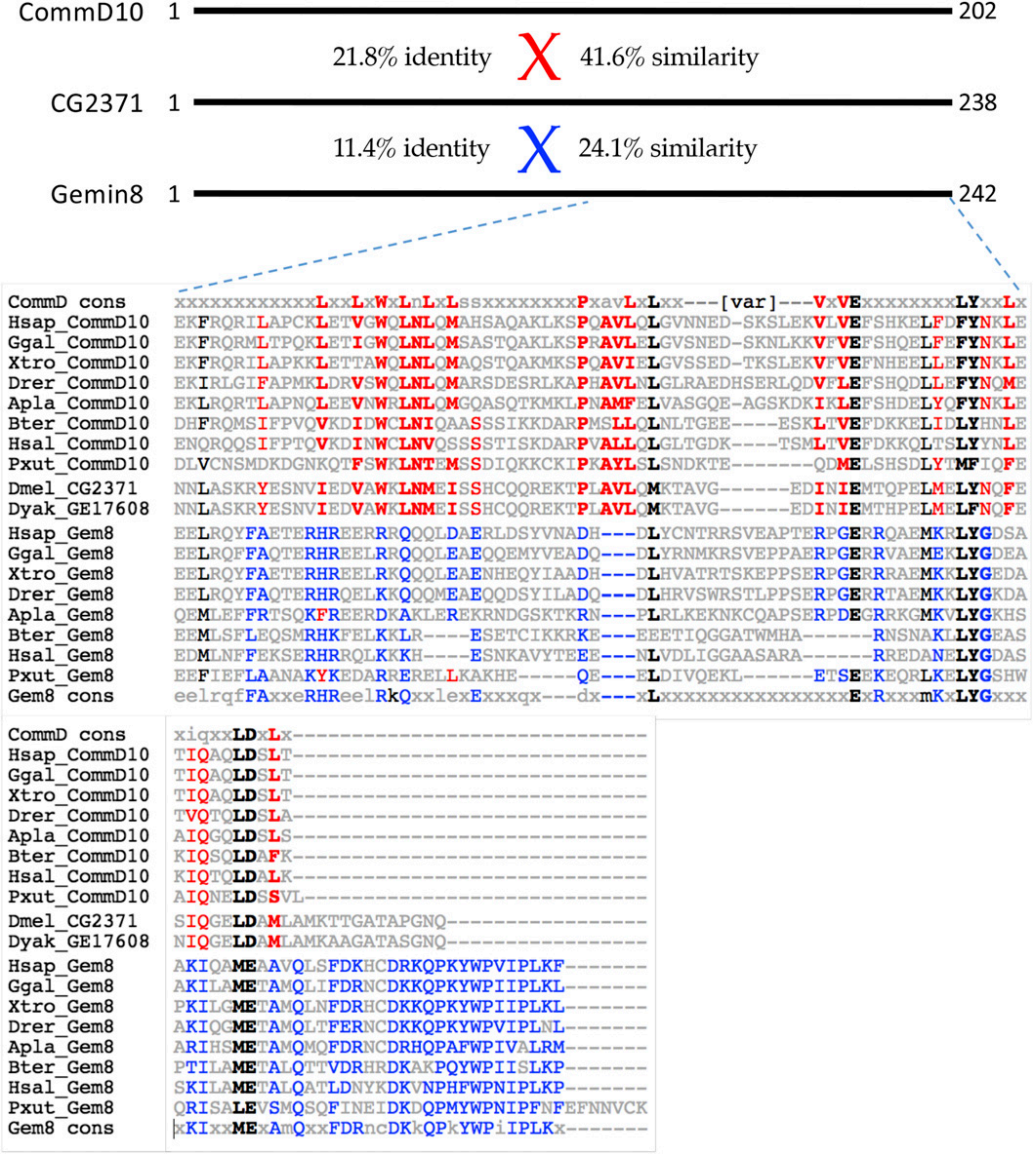
CG2371/Valette is orthologous to CommD10, not Gemin8. Top: Schematic of human CommD10 and Gemin8, with pairwise alignment scores to CG2371 showing %similarity and %identity. Bottom: Amino acid alignment of the C-terminal domains of CommD10 and Gemin8 (Gem8) from a variety of metazoan species, including: *Homo sapiens* (Hsap), *Gallus gallus* (Ggal), *Xenopus tropicalis* (Xtro), *Danio rerio* (Drer), *Acanthaster planci* (Apla), and *Bombus terrestris* (Bter), *H saltator* (Hsal), and *Papilio xuthus* (Xut). Two orthologous fruitfly proteins from *D. melanogaster* (Dmel_CG2371) and *D. yakuba* (Dyak_GE17608) are shown for comparison. Diagnostic residues shaded in red text are conserved among CommD10 sequences whereas the blue residues are conserved in Gemin8. For additional comparison, consensus sequences for the C-terminal Comm domain (CommD cons) and C-terminus of Gemin8 (Gem8 cons) are shown at the top and bottom of the alignment, respectively.

Similar to the situation with Gemin6 and Gemin7, we found that CG2371, identified by Lanfranco *et al.* (2017) as the potential Gemin8 ortholog, is more closely related to a protein called CommD10 (Figure 4). In humans, there are ten Comm domain paralogs, five of which are conserved in insects (Maine and Burstein 2007). These proteins are characterized by a conserved C-terminal ∼80 aa region called the Comm domain (see discussion below). Gemin8 orthologs are also conserved at their C-termini, but the structure and function of this protein is largely unknown. As shown in Figure 4, *D.melanogaster* CG2371 and *D.yakuba* GE17608 proteins are most similar to CommD10 orthologs as compared to the Gemin8 orthologs from a variety of metazoan species. The conservation of diagnostic amino acid residues between CG2371 and CommD10 (Figure 4, shaded residues) leaves little doubt as to the ancestral relationship. Again, the interesting question of whether *Valette/CG2371* might have compensated for loss of *Gemin8* within the *Drosophila* lineage is discussed below.

### Gaulos (CG2941) is orthologous to metazoan Gemin4

In contrast to Gem6-7-8, Gemin4 was originally thought to be lost from insects entirely (Kroiss *et al.* 2008), however Lanfranco *et al.* (2017) identified *CG2941/Gaulos* as a potential *Gemin4* ortholog. To investigate this issue, we carried out PSI-BLAST (Position-Specific Iterative Basic Local Alignment Search Tool) analysis (Altschul *et al.* 1997). Using vertebrate Gemin4 proteins as seed sequences, this procedure readily identified several candidates among the Hymenoptera, but not within the Diptera (or it may take more than six iterations to converge). Anecdotally, we have found that the entire SMN complex is well preserved in many Hymenopteran genomes, and so we used the putative Gemin4 sequence XP_003401506.1 from *Bombus terrestris* (Buff-tailed bumble bee) as the starting point for PSI-BLAST. We found that this seed sequence readily identified both vertebrate and invertebrate orthologs, along with three nearly identical *D. melanogaster* proteins, including CG2941, CG32783 and *CG32786*. An alignment of a subset of these identified proteins is presented in Fig. S1. As shown, the overall conservation of Gemin4 is rather modest. For comparison, the putative Gemin4 ortholog (GH21356) from a distantly related fruitfly, *D. grimshawi*, is also shown. Notably, an analysis of the predicted secondary structure of Gemin4 orthologs shows a high degree of conservation (see Fig. S1). Thus, despite the fact that the three *D. melanogaster* proteins (CG2941, *CG32786* and CG32783) are most closely related to metazoan Gemin4, it is difficult to assign orthology on the basis of amino acid conservation alone.

Importantly, Lanfranco *et al.* (2017) did not base their conclusions solely on bioinformatics; these authors also showed that CG2941 interacts with Gemin3 in a targeted genetic modifier screen. Flies expressing low-levels of a dominant-negative *Gemin3* construct lacking its N-terminal helicase domain, called *Gemin3^BART^* (Borg *et al.* 2015), were used in combination with a deficiency allele *Df(1)ED6716* (Ryder *et al.* 2007) that spans the 3F2-4B3 interval on the X chromosome that includes *CG2941/Gaulos* (Lanfranco *et al.* 2017). Loss of one copy of this region in combination with pan-muscular expression of *Gemin3^BART^* led to a marked age-dependent enhancement of the phenotype (Lanfranco *et al.* 2017). These results were encouraging because Gemin4 was originally identified as a putative Gemin3 cofactor (Charroux *et al.* 2000), and so a reduction in *Gemin4* gene copy-number might reasonably be expected to enhance the phenotype of a *Gemin3* hypomorph.

**Figure 5:**
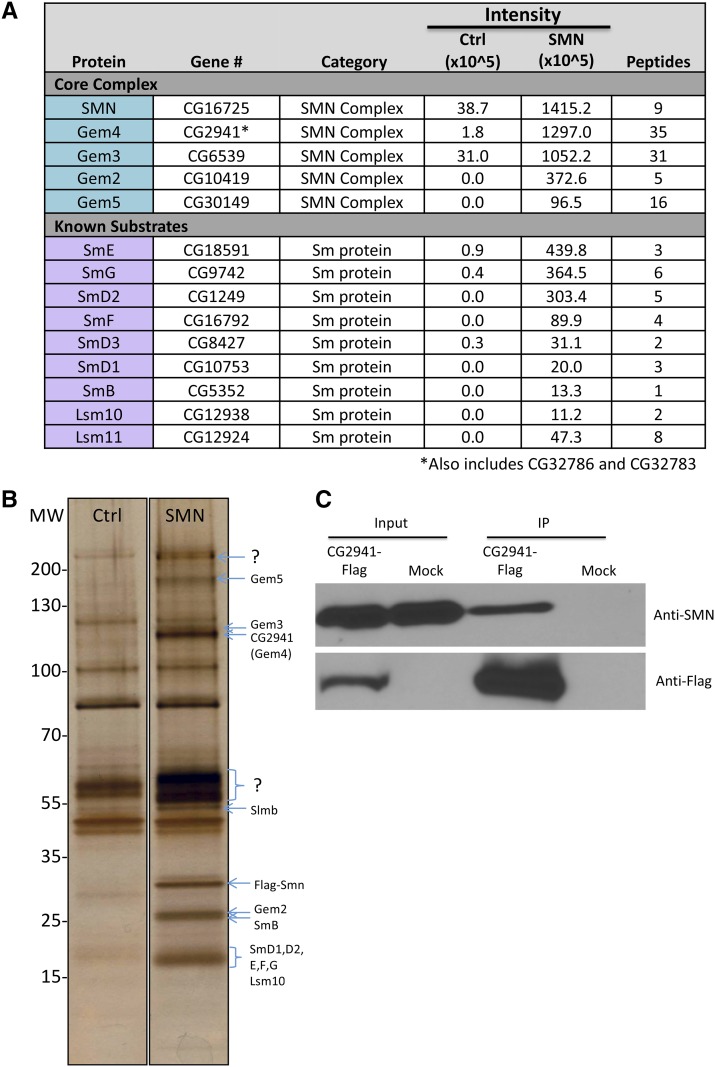
Flag-SMN immunopurified lysates contain members of the known SMN complex and known substrates. (A) Flag-purified eluates were analyzed by label-free mass spectrometry. Proteins in the core SMN complex and known substrates were highly enriched in the SMN sample as compared with Ctrl. (B) Lysates from Oregon-R control (Ctrl) *Drosophila* embryos and those that exclusively express Flag-SMN (SMN) were immunopurified. Protein eluates were separated by SDS-PAGE and silver stained. Band identities were predicted by size. (C) Following transfection of CG2941-Flag in *Drosophila* S2 cells and immunoprecipitation (IP) of total cell lysates with anti-Flag beads, western analysis was carried out using anti-SMN antibodies. Mock transfected cells were analyzed in parallel. Co-purification of endogenous SMN was detected in the CG2941-Flag IP lane but not in the control lane (Mock).

Furthermore, a systematic coaffinity purification analysis of the *Drosophila* proteome showed that CG2941 is capable of forming a complex in S2 cells transfected with HA-tagged SMN (Guruharsha *et al.* 2011). Similarly, co-transfection of S2 cells with HA-tagged SMN and GFP-tagged CG2941/Gaulos confirmed this interpretation (Lanfranco *et al.* 2017). Previously, we carried out proteomic profiling of embryonic lysates from transgenic flies expressing Flag-SMN (Gray *et al.* 2018). Notably, these animals express SMN protein from the native *Smn* control regions in an otherwise null background (Praveen *et al.* 2012; Gray *et al.* 2018). In order to conclusively determine whether CG2941 forms a complex with SMN under endogenous conditions, we directly analyzed eluates of this purification by label-free mass spectrometry (Figure 5A). We also carried out SDS-PAGE analysis of the purified samples, followed by silver staining (Figure 5B). In addition to identifying all of the known Sm protein substrates of SMN, we also identified Gemin2, Gemin3, Gemin5 and CG2941 as highly-enriched SMN binding partners (Figure 5). Notably, mass spectrometry failed to detect CG14164 (Hezron/Lsm12b), CG31950 (Sabbat/Naa38) or CG2371 (Valette/CommD10) among the co-purified proteins (see below for a discussion).

We also note that *CG32786* and CG32783 are so similar to CG2941 that most of their tryptic peptides are indistinguishable from one another. However, we identified 35 peptides corresponding to these three proteins and their overall enrichment in the purified eluates was comparable to that of the other core members of the SMN complex (Figure 5A). To confirm this interaction, we performed a reciprocal coimmunoprecipitation analysis with CG2941-Flag in S2 cells. As shown in Figure 5C, CG2941-Flag also co-purifies with endogenous SMN. On the basis of these findings, we conclude that *CG2941/Gaulos* is indeed orthologous to human *Gemin4*.

### CG2941 is ancestral to CG32786 and CG32783 and is part of a genomic triplication

**Figure 6:**
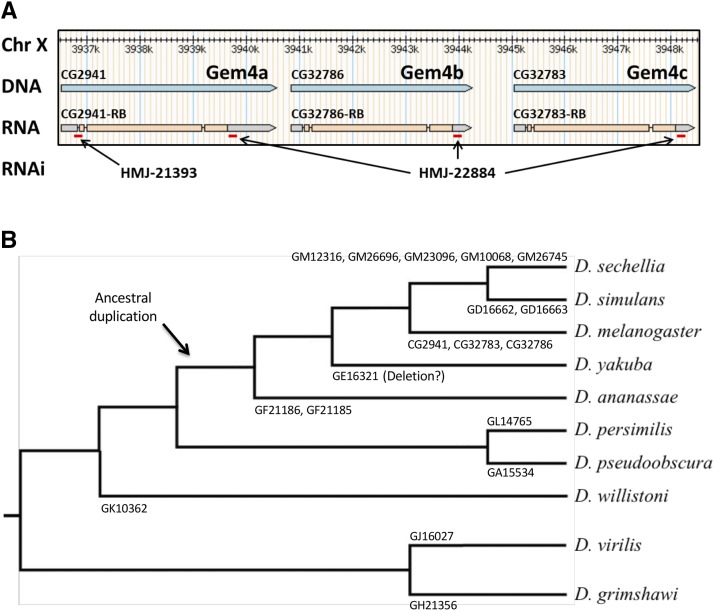
Organization and phylogeny of Gemin4/CG2941-like genes in Drosophilids. (A) Browser shot of *D. melanogaster* CG2941 paralogs, showing their relative location and exon-intron structure. Red dashes indicate regions targeted by short hairpin (sh)RNAs expressed from Gal4-inducible RNAi constructs, see text for details. (B) *Drosophila* phylogenetic tree showing gene name and number of respective Gemin4 (Gem4) orthologs within each lineage. Following their divergence from the obscura group (*D. pseudoobscura* and *D. persimilis*), potential duplications and deletions within the melanogaster group (*D. sechellia*, *D. melanogaster*, *D. simulans*, *D. yakuba*, *and D. annanasae*) are illustrated.

As shown in Figure 6A, *CG2941*, *CG32786* and *CG32783* are tightly linked in the 3F7-3F9 interval on the *D. melanogaster* X chromosome. A comparison of their DNA sequences reveals that these three genes are extremely similar, with *CG32786* and *CG32783* being more closely related to each other than they are to *CG2941*. In contrast, *CG2941* shares more sequences with the orthologous sequences in other more distantly related Drosophilids than do *CG32786* or *CG32783*. Thus, we infer that *CG2941* is ancestral to the *CG32786* and *CG32783* gene pair.

Interestingly, this region of the genome appears to be somewhat fluid, particularly within the melanogaster group, which includes *D. sechellia*, *D. melanogaster*, *D. simulans*, *D. yakuba*, *and D. annanasae*. Phylogenetic analysis of the number of *CG2941*-like genes in various Drosophilid genomes (Figure 6B) suggests that an ancestral duplication of *CG2941* occurred sometime between the divergence of the melanogaster group and the obscura group, the latter of which contains *D. pseudoobscura* and *D. persimilis*. The hawaiian (represented by *D. grimshawi*) and virilis (*D. virilis*) groups each only have one *CG2941*-like gene and thus serve as outgroups for this analysis. Within the melanogaster group there appears to be ongoing genetic rearrangement of this genomic region, as certain species have up to five different copies of this gene, whereas others have only a single copy (Figure 6B). For ease of future identification, we suggest the following nomenclature for the *D. melanogaster* genes: *Gemin4a (CG2941/Gaulos)*, *Gemin4b (CG32786) and Gemin4c (CG32783)*.

### Gemin4 gene function is essential for viability in Drosophila

The FlyAtlas anatomical and developmental expression database (Chintapalli *et al.* 2007; Leader *et al.* 2018) shows that *CG2941* is expressed ubiquitously, albeit at relatively low levels. Its highest levels of expression are in the larval central nervous system and the adult ovary. Because the sequences of the three *Gemin4* paralogs are so similar, the function and expression levels of the other two genes are unclear. Lanfranco *et al.* (2017) employed an RNA interference transgene targeting *CG2941*, but did not mention the existence of the other two paralogous genes in their publication. We note that this transgene (VDRC 52356), obtained from the Vienna Drosophila Resource Center, expresses a 339bp dsRNA that targets all three *Gemin4* paralogs (*CG2941*, *CG32786* and *CG32783*). Furthermore, the deficiency, *Df(1)ED6716*, used in the original genetic interaction screen also uncovers all three paralogs.

To confirm and extend these studies, we sought to determine whether or not loss of *CG2941* might be compensated by the presence of the other paralogs. We therefore carried out RNAi using two different shRNA expressing TRiP lines obtained from the Transgenic RNAi Project (Perkins *et al.* 2015). As shown in Figure 6A, *HMJ-21393* specifically targets a region near the 5′-end of the *Gemin4a/CG2941* transcript, whereas the *HMJ-22884* construct targets a 3′-UTR sequence that is shared by all three transcripts.

**Figure 7:**
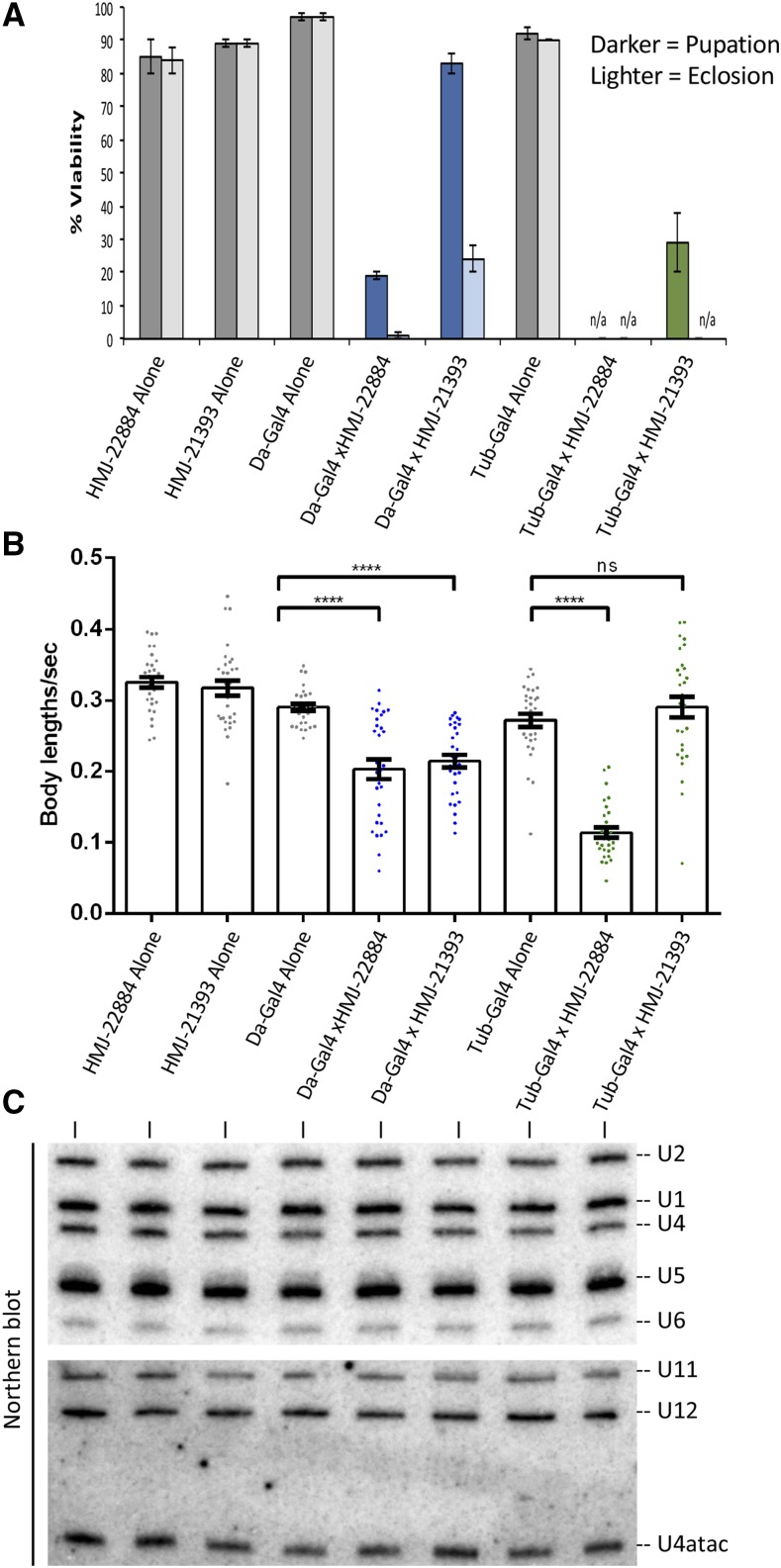
*CG2941* and its paralogs are essential for viability and locomotion independent of snRNP biogenesis. (A) *daughterless* (Da) or *tubulin* (Tub) Gal4 drivers were crossed to RNAi lines specifically targeting *CG2941* (HMJ-21393) or all three paralogs (HMJ-22884). 100 larvae per cross were selected and viability was measured based on the number of larvae that pupated (darker bars) and that eclosed (lighter bars). “n/a” represents a category in which there were no pupae (pupation) or adults (eclosion) to count. Error bars show standard error of the mean. (B) Wandering third instar larvae from the same crosses in (A) were recorded and locomotor behavior was measured using the wrMTrck plug-in for Fiji/ImageJ. Error bars represent standard error. (C) Early third instar larvae (73-77 hr post-egg laying) from the same crosses in (A) were collected and RNA was extracted to measure snRNA levels via northern blotting. See Fig. S2 for details on the knockdown. Probe sequences are described in Materials and Methods.

In Figure 7A, we used the Gal4/UAS system to drive these two UAS-shRNA constructs ubiquitously using either *daughterless*-Gal4 (Da-Gal4) or *tubulin*-Gal4 (Tub-Gal4). Expression of either drivers or the responders alone had little to no effect on organismal viability (Figure 7A). However, ubiquitous expression of the shRNA that targets all three genes (*HMJ-22884*) was essentially larval lethal (Figure 7A). The phenotype of animals expressing the *HMJ-21933* construct that specifically targets *CG2941* was slightly less severe, as most of the animals complete larval development (*Da-Gal4* x *HMJ-21933*), and roughly 20% of them eclose as adults. The phenotype of the *Tub-Gal4* x *HMJ-21933* cross was even more severe, as only ∼30% of the animals reached pupal stages and none progressed to adulthood (Figure 7A). These findings are consistent with those in mice (Meier *et al.* 2018), showing that *Gemin4* is an essential gene. The data also suggest that *Gemin4b/CG32786* and *Gemin4c/CG32783* can partially compensate for loss of *Gemin4a/CG2941*. However, in the absence of specific genetic lesions and complementation analysis, it is difficult to make firm conclusions.

To investigate the consequences of Gemin4 loss of function, we carried out larval locomotion assays and northern blotting analysis. Wandering third instar larvae were used to record their movement on a molasses agar plate. The videos were then converted and analyzed using the wrmTrck plug-in of Fiji/ImageJ to generate a measurement of body lengths per second (BLPS), which takes into account the speed and size of each larva. For northern blotting, total RNA was harvested from early third instar (72-76hr) larvae, just prior to the beginning of the lethal phase of the RNAi. As shown in Figure 7C, we did not detect signficant reductions in the levels of either the major (U1, U2, U4 and U5) or the minor (U11, U12 and U4atac) Sm-class snRNAs. Due to the long half-lives of spliceosomal snRNPs in cultured mammalian cells (1-3 days, depending on the snRNA; Sauterer *et al.* 1988), this finding is perhaps not so surprising. Thus the presumptive loss of Gemin4 function in snRNP biogenesis may not have had time to affect these animals. Given that complete loss of SMN protein only results in a ∼50–60% reduction of U1-U5 snRNAs at this stage of development (Garcia *et al.* 2016), it is also unsurprising that knockdown of Gemin4 has a less dramatic effect. We conclude that the larval lethality associated with *Gemin4* loss of function is not due to a concomitant loss of snRNPs.

### Conservation of Gemin4 among Dipteran genomes

In the ten years since Kroiss *et al.* (2008) first suggested that Gemin4 was missing from the Dipteran SMN complex, there have been numerous hints to the contrary. As early as 2009, raw mass spectrometry data released by the DPiM (*Drosophila* Protein interaction Map) project showed that CG2941 co-precipitates with ectopically expressed, epitope-tagged SMN in S2 cells (https://interfly.med.harvard.edu). Subsequent quality control steps apparently removed CG2941 from the list of potential SMN interactors despite the fact that it also co-purifies with epitope tagged Gemin2 and Gemin3 (Guruharsha *et al.* 2011; Guruharsha *et al.* 2012). On the basis of biochemical purifications from fly embryos and S2 cells, we and others have speculated that CG2941 might well be a *bona fide* core member of the SMN complex (Sen *et al.* 2013; Gray *et al.* 2016). However, in the absence of additional genetic, phylogenetic and biochemical evidence linking endogenous CG2941 to SMN, the conservation of Gemin4 remained an open question.

Three new lines of experimentation demonstrate that *CG2941* is indeed *Gemin4*. First, Lanfranco *et al.* (2017) found that an N-terminally truncated *Gemin3^∆N^* construct interacts genetically with a deficiency that uncovers *CG2941/Gemin4a*, *CG32786/Gemin4b* and *CG32783/Gemin4c*. They also showed that RNAi-mediated knockdown of all three genes enhanced the phenotype of this dominant negative *Gemin3^∆N^* transgene. Second, ongoing metazoan genome sequencing efforts allowed us to more confidently predict *Gemin4* orthologs on the basis of primary sequence (Fig. S1). Third, we found that endogenous CG2941, *CG32786* and CG32783 co-purify with SMN expressed from its native promoter *in vivo* in fly embryos (Figure 5). The relative enrichment and number of peptides corresponding to CG2941 in the mass spectrometry experiment was similar to that of the other Gemins (Figure 5A). These findings lead us to conclude that *Gemin4* has been retained in the genomes of *Drosophila* and other dipterans.

### SMN and the evolution of Gemin subcomplexes

The human SMN complex can be subdivided into several distinct subunits (Battle *et al.* 2007; Otter *et al.* 2007). SMN and Gemin2 form an oligomeric heterodimer (SMN•Gemin2)_n_ that makes up the core of the complex (Fischer *et al.* 1997; Gupta *et al.* 2015). Gemin3 binds directly and independently to Gemin4, tethering them both to SMN•Gemin2 (Charroux *et al.* 1999; Charroux *et al.* 2000). Oligomerization of SMN appears to be required for Gemin3 to enter the complex (Praveen *et al.* 2014). Gemin5 is a large (175 kD) WD-repeat protein that recruits RNA substrates to the SMN complex (Battle *et al.* 2006b) via subdomains that bind to the m7G-cap and Sm-site, respectively (Xu *et al.* 2016). Thus Gemin5 can be viewed as an RNP subunit of the SMN complex. Finally, Gemin6 and Gemin7 heterodimerize (Baccon *et al.* 2002; Ma *et al.* 2005) and recruit Gemin8 (Carissimi *et al.* 2006) to form a Gem6-7-8 subunit, the function of which is unknown. As shown in Figures 2 and 3, these three proteins appear to have been lost from Drosophilids, but retained in the genomes of other insects.

An important question raised by our findings is whether or not the functions normally carried out by *Gemin6, Gemin7* and *Gemin8* may have been taken over by *CG14164 (Hez/Lsm12b)*, *CG31950 (Sbat/Naa38)* and *CG2371 (Vlet/CommD10)*. Lanfranco *et al.* (2017) showed that these three proteins are each capable of forming complexes with exogenously expressed SMN when they are transfected into S2 cells. And given the opportunity to interact in a directed yeast two-hybrid screen, Sbat/Naa38 scored positively for interaction with Gemin3, Hez/Lsm12b and Vlet/CommD10 (Lanfranco *et al.* 2017). Interestingly, both Hez and Sbat are predicted contain an Sm-fold, also known as a small beta barrel (Youkharibache *et al.* 2019). This structure is characterized by five short beta strands that form a closed domain wherein the first strand is hydrogen bonded to the last (Arluison *et al.* 2006).

Small beta barrel containing proteins exhibit a strong tendency to form higher-order structures, as exemplified by the Sm and Lsm proteins, found in all three domains of life (Youkharibache *et al.* 2019). Thus, despite the fact that *Hez*, *Sbat* and *Vlet* are not orthologous to *Gemin6*, *Gemin7* and *Gemin8*, respectively, it remains formally possible that they have been evolutionarily co-opted into the SMN complex in flies. Our inability to identify these three proteins as endogenous SMN binding partners by mass spectrometry (Figure 5) argues against this idea. However, stable protein interactions are not required to elicit important biological outcomes, so additional experiments will be needed to conclusively demonstrate a role for Hez/Lsm12b, Sbat/Naa38 and Vlet/CommD10 in SMN biology.

### Considerations and Prospects

In the mean time, several interesting possibilities suggest themselves for future investigation. We and others have hypothesized that the SMN complex may function as a hub for various cellular signaling pathways, in addition to its role in chaperoning snRNP biogenesis (Raimer *et al.* 2017; Gray *et al.* 2018 and references therein). As shown in Figure 4, the fruitfly *CommD10* ortholog is *Vlet/CG2371*. Intriguingly, human CommD (Copper metabolism Murr1 domain) proteins can form homo- and hetero-dimers (Burstein *et al.* 2005) and are involved in a variety of cellular pathways including endosomal membrane trafficking and the inhibition of NF-kB signaling (Bartuzi *et al.* 2013; Mallam and Marcotte 2017). Both SMN (Kim and Choi 2017) and Gemin3 (Shin *et al.* 2014) have been implicated in NF-kB related pathways. It is tempting to speculate that human Gemin8 might play a role in linking the SMN complex to NF-kB signaling by interacting with, or otherwise functioning as, a CommD-like protein. Given the potential for Sbat/Naa38 to interact with Gemin3 (Lanfranco *et al.* 2017), perhaps the Gem6-7-8 subcomplex functions as a regulatory subunit that modulates the activity of SMN and/or Gemin3.

Irrespective of any putative role in cellular signaling, the fact that Sbat/Naa38 contains an Sm-fold may help to explain several interesting observations in the literature. Metazoan Naa38 (a.k.a. Lsmd1, Mak31) is an auxiliary subunit of NatC (N-terminal acetyltransferase C; Starheim *et al.* 2009). N(alpha)-acetyltransferases are enzymes that consist of a catalytic subunit and one or two auxiliary subunits (Aksnes *et al.* 2016). The auxiliary subunits modulate the activity and substrate specificity of the catalytic subunit. Furthermore, they mediate co-translational binding to the 60S ribosome, in a region that is located near the nascent polypeptide exit tunnel (reviewed in Aksnes *et al.* 2016). This latter point merits attention for two reasons.

First, Fischer and colleagues recently reported data suggesting that, following their translation, Sm proteins can remain bound to the ribosome near the exit tunnel, dissociating only after binding to the assembly chaperone pICln (Paknia *et al.* 2016). These authors hypothesize the existence of a quality control hub for chaperone-mediated protein assembly, located on the ribosome. Whether or not Sm protein heterodimers (*e.g.*, Lsm10/11 and SmD1/D2) actually bind to the nascent peptide tunnel region of the ribosome *in viv*o is unclear. However, the fact that metazoan Naa38 is structurally similar to Sm proteins provides a plausible mechanism for their binding to the ribosome immediately following translation. Given that Gemin6 and Gemin7 are also members of the Sm-like superfamily of proteins (Ma *et al.* 2005) it is conceivable that Sbat/Naa38 could dimerize with other Sm-like proteins (*e.g.*, Hez/Lsm12b) in *Drosophila*.

Second, Naa38 might not be a *bona fide* member of the SMN complex (in flies or any other species), but it could potentially interact with SMN as part of its *canonical* function in N-terminal protein acetylation. More than 80% of human proteins are co-translationally modified on their N-termini (Arnesen *et al.* 2009), however the functional impact of this modification is largely unknown. Most proteins do not retain their N-terminal Met residue, and its removal by methionine aminopeptidases frequently leads to acetylation of the resulting N-terminus, particularly if the second residue is Ala, Val, Ser, Thr or Cys (Hwang *et al.* 2010). Interestingly, the N-terminal Ala2 residue of SMN is known to be acetylated in human cells (Van Damme *et al.* 2012), and an A2G missense mutation in the *SMN1* gene is known to cause a mild form of SMA when *SMN2* is present in a single copy (Parsons *et al.* 1998). This mutation is puzzling because, with the exception of Ala2, the N-terminal 15aa of SMN (*i.e.*, upstream of the Gemin2 binding domain) are very poorly conserved. Moreover, changing Ala2 to Gly is predicted to reduce the probability of N-terminal acetylation and recognition by the N-end-rule proteasomal degradation pathway (Hwang *et al.* 2010). These findings suggest that the phenotype of the A2G mutation in humans is due to loss of N-terminal acetylation of SMN.

In conclusion, it seems unlikely that three different proteins (CG14164, CG31950, CG2371) derived from three different biological contexts might be co-opted into a novel Gemin subcomplex. A loss of the Gem6-7-8 subunit from the SMN complex in flies would suggest that either this subunit is not essential for basal metazoan viability or that other factors have compensated for deficiency of these proteins in *Drosophila*. Additional experiments will be needed to rule in, or rule out, any such functional adaptation. In contrast, the identification of *Gemin4* via PSI-BLAST in a variety of different insect genomes including the Diptera, Hemiptera, Lepidoptera, and Hymenoptera (this work) indicates that this protein is widely conserved. Moreover, genetic loss of function studies (Figure 7; Lanfranco *et al.* 2017; Meier *et al.* 2018) strongly suggest that *Gemin4* is essential for metazoan viability.
